# Sequence Determinants of Substrate Ambiguity in a HAD Phosphosugar Phosphatase of *Arabidopsis Thaliana*

**DOI:** 10.3390/biology8040077

**Published:** 2019-10-09

**Authors:** José A. Caparrós-Martín, Iva McCarthy-Suárez, Francisco A. Culiáñez-Macià

**Affiliations:** 1 Instituto de Biología Molecular y Celular de Plantas ‘‘Eduardo Primo Yúfera’’ (UPV-CSIC), Universidad Politécnica de Valencia, Ciudad Politécnica de la Innovación (CPI), C/ Ingeniero Fausto Elio s/n, ES-46022 Valencia, Spain; ivmcsua@upvnet.upv.es (I.M.-S.); faculia@ibmcp.upv.es (F.A.C.-M.); 2 CHIRI Research Centre, Faculty of Health Sciences, School of Pharmacy and Biomedical Sciences, Curtin University, Perth 6102, WA, Australia

**Keywords:** *Arabidopsis*, HAD superfamily, hydrolases, sugar phosphatases, promiscuity

## Abstract

The *Arabidopsis thaliana* broad-range sugar phosphate phosphatase AtSgpp (NP_565895.1, locus AT2G38740) and the specific DL-glycerol-3-phosphatase AtGpp (NP_568858.1, locus AT5G57440) are members of the wide family of magnesium-dependent acid phosphatases subfamily I with the C1-type cap domain haloacid dehalogenase-like hydrolase proteins (HAD). Although both AtSgpp and AtGpp have a superimporsable *α*/*β* Rossmann core active site, they differ with respect to the loop-5 of the cap domain, accounting for the differences in substrate specificity. Recent functional studies have demonstrated the essential but not sufficient role of the signature sequence within the motif-5 in substrate discrimination. To better understand the mechanism underlying the control of specificity, we explored additional sequence determinants underpinning the divergent evolutionary selection exerted on the substrate affinity of both enzymes. The most evident difference was found in the loop preceding the loop-5 of the cap domain, which is extended in three additional residues in AtSgpp. To determine if the shortening of this loop would constrain the substrate ambiguity of AtSgpp, we deleted these three aminoacids. The kinetic analyses of the resulting mutant protein AtSgpp3Δ (ΔF53, ΔN54, ΔN55) indicate that promiscuity is compromised. AtSgpp3Δ displays highest level of discrimination for D-ribose-5-phosphate compared to the rest of phosphate ester metabolites tested, which may suggest a proper orientation of D-ribose-5-phosphate in the AtSgpp3Δ active site.

## 1. Introduction

Molecular biology has evolved from the central dogma of gene–protein–specific function [1,2] and the analogy of specific substrates suiting the binding pocket lock [3]. Thus, substrate specificity is considered the consequence of the divergent evolutionary selection exerted on the substrate promiscuity activity of precursor enzymes [4,5]. The ability of enzymes to accommodate different substrates is known as substrate ambiguity [6]. Substrate ambiguous enzymes require active site plasticity [7,8] and the involvement of additional functional residues besides those essential for core catalytic activity [9,10,11,12,13].

Catalytic promiscuity and substrate ambiguity are keys to evolvability, which in turn is pivotal to the successful acquisition of novel biological functions [14]. In an earlier work [15], we investigated the determinants of substrate promiscuity focusing on the evolutionary divergence of two *Arabidopsis* members of the haloacid dehalogenase-like hydrolase proteins (HAD) superfamily. The members of this family share a number of structural features including the *α/β* core domain catalytic scaffold, in which the active site consists of four loop motifs containing highly conserved residues. Motifs one (DxD), two (T/S) and three (K/R) are less flexible than motif 4, which has been variously described (E/DD, GDxxxD×××D or GDxxxxD). Spatially, these four structural modules are arranged around the substrate-binding groove, which positions in the active site architecture the key residues involved in the core chemistry [16]. Many of the HAD family members also contain a smaller module connected to the core domain by two hinge loops. This module known as cap domain, acts as a lid over the core catalytic domain. The cap domain localizes residues that are important for the enzymatic catalysis such as those contained in loop-5. This loop locates a stringently conserved Gly residue flanked by amino acids whose side chains contribute to the catalytic site formed at the domain–domain interface. While the α/*β* Rossmann fold structural domains are superimposable, the configuration of the cap domain is less conserved even considering the closest structural homologs [17].

*Arabidopsis thaliana* AtGpp (NP_568858.1) [18] and AtSgpp (NP_565895.1) [19] are both HAD-type phosphosugar phosphatases. Structural prediction and chemistry analysis classify them as typical phosphomonoesterases of subclass I (C1-type cap) [19]. Although both enzymes share significant similarity (39%, sequence identity of 23%) and their predicted tertiary structures are superimposable, their substrate specificity is different. AtGpp exhibits a specific preference for the hydrolysis of DL-glycerol-3-phosphate, while AtSgpp is a promiscuous enzyme that shows phosphate phosphatase activity over a broad-range of sugar substrates, including intermediates of the pentose phosphate and glucose metabolism such as D-glucose-6-phosphate, D-ribose-5-phosphate, D-fructose-6-phosphate, or DL-glycerol-3-phosphate, amongst others [18,19]. The structural motifs of the core catalytic domain of both AtSgpp and AtGpp proteins present the same amino acid composition [16,20]. Conversely, large differences are observed in the residues forming the substrate specificity region (loop-5) of the cap domain [16]. Residues in the loop-5, are responsible for the substrate diversification within the HAD family and provide substrate specificity [20,21]. However, the substitution of the entire motif-5 in AtSgpp (IAGKH) with that of AtGpp (MMGRK) does not restrict AtSgpp activity to the DL-glycerol-3-phosphate [15]. The lack of activity of the resultant AtSgpp mutant enzyme (I68M, A69M, K71R, H72K) lead us to hypothesize a more complex chemistry in which, apart from those of the sequence motif-5, the side chains of other residues of the cap domain would be involved [16]. Superimposition of protein models shows few structural changes between AtSgpp and AtGpp, with exception of a three-residue longest loop upstream of the substrate specificity loop-5 of AtSgpp that extends its cap domain. To test if this geometry represents a sequence determinant that supports the activity of AtSgpp over multiple substrates, we constructed a mutant lacking these additional residues to mimic the cap domain of AtGpp. With the aim of contributing to a better understanding of the divergent evolutionary selection exerted on promiscuous enzymes, the specificity and kinetics of the mutated protein was analyzed and compared with the AtSgpp chemistry.

## 2. Materials and Methods

### 2.1. Materials

The reagents used for cloning were: pMAL-c2x vector and *Escherichia coli* TB1 host for expression (New England Biolabs, Hitchin, Hertfordshire, UK), REDTaq DNA polymerase (Sigma, St. Louis, MO, USA), oligonucleotides (Sigma-Genosys, Gillingham, Dorset, UK), and pBluescript SK+ vector (StrataGene, Kirkland, WA, USA). The pSBETa helper vector was constructed at the Max-Planck Institute (Köln, Germany) [22].

### 2.2. Computational Biology

Comparative analysis was performed using programs such as BLAST [23] and data bank resources from the NCBI. Protein domain families were generated with the ProDom program from the Swiss-Prot and TrEMBL sequence databases [24,25]. CLUSTAL W was used for the progressive multiple sequence alignment [26]. Three-D models of the protein have been built using the ESyPred3D web server [27] and the visualization with the molecular graphics program RasMol [28]. PROPKA was used for the p*K*_a_ predictions [29].

### 2.3. Site-Directed Mutagenesis

AtSgpp mutant was constructed by sequential PCR steps [30], using appropriate mutagenesis primers and a previously generated cassette containing the coding region of the locus *AT2G38740* as template [20]. The primers (1) forward, 5′-CGAGGAATTCATGAATGGCTTCTCTGATCTTAATCC-3′ and (2) reverse, 5′-CCGGGTCGACTTAAGACTTGTTATCAAGTTCTTCC-3′ were used as 5*′*- and 3*′*- terminal primers for the sequential PCR. The PCR product was cloned as 735 bp *Eco* RI/*Sal* I fragment into the pBluescript SK+ vector. Sequencing of the pBluescript SK+ clones revealed that the sequence of the protein was the expected.

The mutant *AtSgpp* gene AtSgpp3Δ (ΔF53, ΔN54, ΔN55) was then subcloned into the single *Eco*RI and *Sal*I sites of the pMAL-c2x expression vector and transformed into the expression strain *E. coli* TB1 for recombinant protein production. The cloning site used in the pMAL-c2x polylinker (locate downstream of the malE gene), adding vector-encoded residues Ile-Ser-Glu-Phe fused between the factor xa cleavage site and the NH_2_-terminal methionine residue of the cloned proteins. To improve the expression of the eukaryotic genes in the *E. coli* system, *E. coli* TB1 cells were co-transformed with pMAL-c2x AtSgpp or pMAL-c2x AtSgpp3Δ and, in each case, the helper plasmid pSBETa. The positive co-transformed colonies were selected on 200 µg/mL ampicillin and 100 µg/mL kanamycin (Sigma, St. Louis, MO, USA).

### 2.4. Purification of Recombinant Proteins

Selected co-transformed *E. coli* strains, containing fusion plasmid pMAL-c2x AtSgpp, or pMAL-c2x AtSgpp3Δ and, in each case, the helper pSBETa, were grown at 37 °C to 2 × 10^8^ cells/mL (A_600_~0.5) in 1 L of rich broth + glucose and ampicillin + kanamycin (10 g tryptone, 5 g yeast extract, 5 g NaCl, 2 g glucose, autoclave; adding sterile 200 µg/mL ampicillin, and 100 µg/mL kanamycin), induced with 1 mM isopropyl-*β*-D-thiogalactoside (IPTG) (Ambion, Austin, Tx, USA), and harvested 2 h after induction. Fusion proteins were released from the harvested cells by sonication in column buffer (20 mM Tris-HCl pH 7.4, 200 mM NaCl, and 1 mM EDTA), collected after elution from the amylose resin (New England Biolabs, Hitchin, Hertfordshire, UK) with column buffer +10 mM maltose (Sigma, St. Louis, MO, USA) and the concentration determined by the Bradford method [31]. Proteins were separated by SDS-PAGE electrophoresis in 12% polyacrylamide gels [32], using prestained molecular weight standards (New England Biolabs, Hitchin, Hertfordshire, UK). Both the MBP-AtSgpp wild type and the MPB-AtSgpp3Δ mutant enzymes showed similar expression levels and solubility (Figure S2B).

### 2.5. Activity Assays

The biochemical characterization of the purified enzymes was assayed as previously described [15,18,19,33,34]. The reaction mixture contained 20 mM Tris-HCl pH 7.0, 5 mM MgCl_2_, and 10 mM D-ribose-5-phosphate, 2-deoxy-D-glucose-6-phosphate, D-mannose-6-phosphate, D-glucose-6-phosphate, D-fructose-6-phosphate, or DL-glycerol-3-phosphate (R7750, D8875, M6876, G7250, F1502, G2138 respectively, Sigma, St. Louis, MO, USA). Release of inorganic phosphate (indicative of phosphatase activity) was monitored through the formation of bluish phosphomolybdenum species in the presence of ascorbic acid and molybdate [34] Spectrometry was then used to determine the concentration of phosphate in the reaction mix. The Michaelis–Menten kinetic parameters *K*_m_ and *V*_max_ were estimated from the activity of the enzyme over different substrate concentrations. The influence of pH in the catalytic activity of the enzyme was determined using a pH range from 2–10 as previously described [15,19]. The experiments were performed at least twice with replicate values showing low inter-variability (estimated from a coefficient of variation lower than 5%).

## 3. Results

### 3.1. Sequence and Structure Comparisons

**Figure 1:**
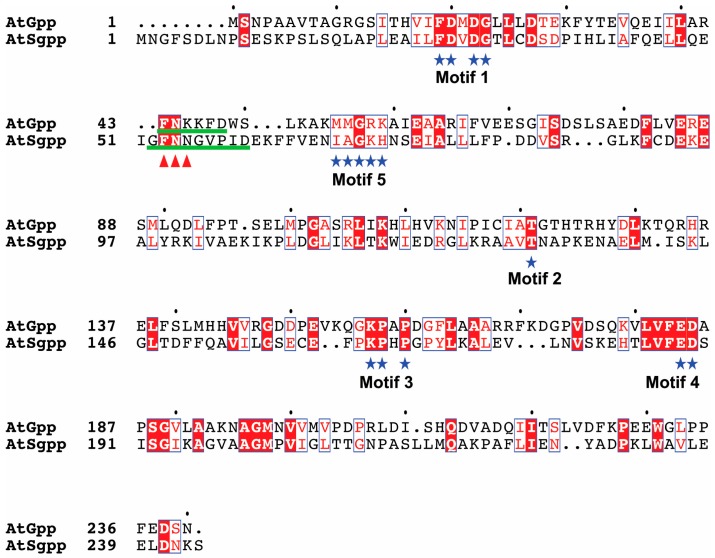
Comparison of the global structure arrangement of the *Arabidopsis* AtSgpp and AtGpp proteins. Conserved amino acids are shown in red on white background. Identical amino acids are depicted in white on red background. The residues contained in the structural elements are indicated with a blue star. Residues in the cap module taking part of the active site, the boundaries of the cap sequence region defined by motifs 1 and 2, and the conserved Gly (G) that identifies the motif 5 motif within this segment, were derived from the information of the predicted 3D models. The loop preceding the motif-5 is underlined in green. The deleted residues in AtSgpp are marked with red arrowheads. This figure was generated using ESPript [37].

Figure 1 shows the significant similarity (39%, sequence identity of 23%) shared between the *A. thaliana* phosphosugar phosphatase AtSgpp [19] and DL-glycerol-3-phosphatase AtGpp [18]. The expected structure classified both proteins as members of the HAD wide family of magnesium-dependent acid phosphatases subfamily I with the C1-type cap domain [19,35]. The greatest similarity occurs at the four motifs forming the catalytic scaffold of the active site platform framed by the core domain [36], which contains the highly conserved sequence motifs (selfsame in both proteins) by which family members are recognized (Figure 1, motifs 1, 2, 3, and 4). These motifs position substrate-cofactor-binding and catalytic residues that are involved in the core chemistry [16]. Conversely, apart from the stringently conserved loop marker Gly (G) flanked by Lys/Arg (K/R), limited sequence homology is shared at the predicted cap domain substrate recognition motif 5, which is responsible for the chemical diversification within the family. Motif-5 positions residues whose side chains contribute to the catalytic site, probably operating in domain–domain binding, active-site desolvation, and/or catalysis [20] (Figure 1). Apart from sequence homology, AtSgpp and AtGpp show overlapping structures. The more shocking difference is observed in the loop preceding the motif 5 of the cap domain, which is predicted to be extended by three additional residues in AtSgpp (Figure 1). To verify whether these differences within the cap domain could be related to the different substrate affinity shown by both enzymes, we investigated the activity of the AtSgpp mutant AtSgpp3Δ (ΔF53, Δ N54, ΔN55), which mimics the length of the counterpart loop in AtGpp. The structural model of the resulting mutant AtSgpp3Δ did not distort either the proper predicted folding of the rest of the protein structure (Figure S1), or the p*K*_a_ values of the side chains of catalytic residues in the active site.

### 3.2. Comparison of Kinetic Analysis

As we previously observed [19], AtSgpp shows affinity over a broad-range of sugar phosphate substrates being D-ribose-5-phosphate, 2-deoxy-D-glucose-6-phosphate, and D-mannose-6-phosphate the preferred substrates amongst the phosphosugar species tested in our experimental set up [19]. The substrate specificity of MBP–AtSgpp3Δ was further investigated for phosphatase activity and Pi release, from hydrolyzed substrates under the same conditions, and compared with the normal protein MBP–AtSgpp. The apparent *K*_m_ and *V*_max_ values for phosphoesters/Mg^2+^ were determined by linear regression from spectrophotometric data (Figure S3). In the case of MBP–AtSgpp, the corresponding slopes of the Lineweaver–Burke plot indicated lower *K*_m_/*V*_max_ values for D-ribose-5-phosphate, 2-deoxy-D-glucose-6-phosphate, and D-mannose-6-phosphate, than for D-glucose-6-phosphate, DL-glycerol-3-phosphate, and D-fructose-6-phosphate (Figure S3A). The values of the substrate specificity constant *k*_cat_/*K*_m_ for D-ribose-5-phosphate, 2-deoxy-D-glucose-6-phosphate, D-mannose-6-phosphate, D-glucose-6-phosphate, DL-glycerol-3-phosphate, and D-fructose-6-phosphate are in the range of 2.5–10.7 × 10^3^ M^−1^s^−1^, being the highest *k*_cat_/*K*_m_ value for D-ribose-5-phosphate (10.7 × 10^3^ M^−1^s^−1^) and the lowest for D-fructose-6-phosphate (2.5 × 10^3^ M^−1^s^−1^) (Table 1). Similar to MBP-AtSgpp, the *K*_m_/*V*_max_ value for D-ribose-5-phosphate of MBP–AtSgpp3Δ remained low (Figure S3B). Conversely, MBP–AtSgpp3Δ enzymatic kinetic was progressively affected, particularly for DL-glycerol-3-phosphate (Figure S3B). The *k*_cat_/*K*_m_ for D-ribose-5-phosphate, 2-deoxy-D-glucose-6-phosphate, D-mannose-6-phosphate, D-glucose-6-phosphate, DL-glycerol-3-phosphate, and D-fructose-6-phosphate are in the range of 0.5–8.3 × 10^3^ M^−1^s^−1^, being the highest *k*_cat_/*K*_m_ value for D-ribose-5-phosphate (8.3 × 10^3^ M^−1^s^−1^) and the lowest for DL-glycerol-3-phosphate (0.5 × 10^3^ M^−1^s^−1^) (Table 1).

### 3.3. pH Rate Profile Comparison

The activity of AtSgpp was optimal at neutral pH 7.0 [19]. Noteworthy it is the fact that the activity of MBP-AtSgpp over D-ribose-5-phosphate did not significantly change over the entire pH range, whereas it decreased as pH increased in the case of 2-deoxy-D-glucose-6-phosphate and D-mannose-6-phosphate. On the contrary, MBP-AtSgpp affinity dropped abruptly, following a bell-shaped curve, on D-glucose-6-phosphate, DL-glycerol-3-phosphate, and D-fructose-6-phosphate, particularly at basic pH (Figure S4A). This contrasting activity may arise from different protonation patterns of the residues side chains and/or conformational changes, which may affect substrate binding and/or cap domain closure. Similar activity over the tested substrates was observed for the mutant protein AtSgpp3Δ (Figure S4B).

## 4. Discussion

The infidelities of molecular recognition of enzymes, which generally exert exquisite specificity, serve as evolutionary starting points of evolvability [38]. Enzyme promiscuity is of great importance in evolutionary biochemistry because it provides opportunities for evolution of new functions in nature [39] and conformational variability is one of the structural principles underlying the evolution of new specificities [14]. Following an earlier work [15], in this study we further investigate sequence determinants underpinning substrate ambiguity. For this purpose, we focused on the evolutionary substrate divergence observed in two members of the HAD wide family of magnesium-dependent acid phosphatases subfamily I with the C1-type cap domain in *Arabidopsis*, the broad-range sugar phosphate phosphatase AtSgpp [19] and the specific DL-glycerol-3-phosphatase AtGpp [18]. Both enzymes exhibit the greatest similarity at the four loops forming the catalytic module of the core domain. Sequence alignment shows the residues forming the catalytic domain to be identical, but not the one of cap domain loop-5. The activity of the core chemistry is common to all subfamilies while loop-5 is tailored toward catalysis of the specialized chemistry unique to a subfamily [20]. What is more, the enzymatic activity within the core domain is dependent on the participation of the residues forming the loop-5 of the corresponding cap domain. We showed that substitution of the putative motif-5 (IAGKH) with that of the DL-glycerol-3-phosphatase AtGpp (MMGRK), does not compromise AtSgpp promiscuousness to the specific targeting of DL-glycerol-3-phosphate [15]. This result suggests that substrate discrimination is not uniquely dependent on the sequence of loop-5 [15]. Intrigued by the former result and because the enhanced substrate promiscuity is often not accompanied by significant changes in the active-site region [40], further structural differences were sought in the cap domain that could determine the dissimilar substrate specificities between both phosphatases. The more evident divergence was observed in the loop motif preceding loop-5, which is extended by three additional residues in AtSgpp. As in previous strategies to investigate the structural basis for the differing substrate specificities in phosphate binding motifs [41], a superimposable mutant was constructed to determine whether the shortening of this loop would constrain the folding plasticity and substrate promiscuity of AtSgpp.

As we previously demonstrated, AtSgpp hydrolyzes a wide range of cyclic sugars with noticeable specificity and efficiency (*k*_cat_/*K*_m_ in the range of 2.5–10.7 × 10^3^ M^−1^s^−1^) [19]. Despite this substrate lax specificity, we also observed that AtSgpp could discriminate between isomers that presumably do not accommodate into the active site domain, such as D-glucose-1-phosphate or *α*-D-mannose-1-phosphate [19]. Deletion of AtSgpp residues Phe-53, Asn-54, and Asn-55 does not deprive the phosphatase activity of mutant protein AtSgpp3Δ but alters the kinetics of the mutated enzyme. Thus, the *K*_m_/*V*_max_, *k*_cat_, and *k*_cat_/*K*_m_ values are essentially changed, increasing *K*_m_/*V*_max_ slopes and reducing the value range of turnover rates *k*_cat_ (1.9–3.9 × 10 to 0.7–3.1 × 10 s^−1^) and the substrate specificity constants *k*_cat_/*K*_m_ (2.5–10.7 × 10^3^ to 0.5–8.3 × 10^3^ M^−1^s^−1^), at neutral pH 7.0. It is worth highlighting the low affinity shown for DL-glycerol-3-phosphate (0.5 × 10^3^ M^−1^s^−1^), which might suggest an essential role of the residues in the cap domain Phe-53 and Asn-54 or AtGpp counterparts Phe-43 and Asn-44 in the DL-glycerol-3-phosphate chemistry. Also, the degree of selectivity, defined by the ratio of *k*_cat_/*K*_m_ between two different substrates [42], varied significantly. The AtSgpp3Δ mutant displayed the highest level of discrimination with the D-ribose-5-phosphate versus DL-glycerol-3-phosphate, D-fructose-6-phosphate, D-glucose-6-phosphate, D-mannose-6-phosphate, and 2-deoxy-D-glucose-6-phosphate (*k*_cat_/*K*_m_ ratio equal to 16.6, 6.9, 5.5, 3.0, and 1.7, respectively), compared to the normal protein (*k*_cat_/*K*_m_ ratio equal to 3.5, 4.3, 3.1, 1.6, and 1.5, respectively). Promiscuous substrates are inadequately positioned relative to the catalytic machinery and therefore exhibit low *k_cat_* values [38] and the *k*_cat_/*K*_m_ ratio reflects both substrate binding affinity and proper orientation [42]. Taken together, these data suggest that reduction of cap flexibility in the AtSgpp3Δ mutant may result in an improved substrate-binding affinity and proper orientation of D-ribose-5-phosphate in the active site compared to the other substrates used in this study.

The pH dependence of the *k*_cat_/*K*_m_ is derived from a composite of the ionization of several groups within the substrate and the enzyme [43]. As we previously discussed, in the case of AtSgpp, the pH-dependent reduced activity of this enzyme could be due to differences in side-chain protonation of the residues Lys-71 and His-72 in the cap domain [15,19]. AtSgpp activity over D-ribose-5-phosphate was not significantly affected over the pH range assayed, which suggests a narrower chemoselectivity in the active site for this ligand compared to the remaining substrates tested. Not surprisingly, similar pH activity profile was observed for the mutant protein AtSgpp3Δ. This may be explained because the pH-activity profile is determined by the p*K*_a_ values of the residues in the active site [44], which are not affected by the deletion of Phe-53, Asn-54, and Asn-55 in AtSgpp3Δ.

Substrate ambiguity is a common feature amongst the members of the HAD superfamily, with 75% of the family members being able to metabolize more than five different substrates. Members with the lower degree of structural accessorization of the Rossmann fold (type C0, characterized for having minimal or no cap insertion) exhibit higher substrate specificity than those containing cap domain modules type C1, C2A, or C2B [45]. This observation is somehow intriguing based on the fact that cap domain contains the specificity determinants [20,45]. However, enzymes are more amenable to evolution when the active site is composed of flexible loops, separated from a highly ordered core scaffold [14,46] and accidental hydrogen bonds mediated by water molecules may play a role in promiscuous interactions [38]. Also, the flexibility of short loop regions might be a structural basis for recognizing distinct metabolites and has proved to be essential for discriminating structurally similar substrates [47]. Moreover, behind the broad substrate specificity of the Human Nudix Hydrolase MTH1, is the substrate-dependent shift in the protonation state of neighboring aspartic residues (Asp119 and Asp120) buried in the ligand-binding site [48], as they are Asp28 and Asp30 at AtSgpp motif 1. On this basis, the AtSgpp cap domain could be presumed as the molecular regulator that pivots the active site loop-5 motion to the particular orientation that would occupy diverse phosphosugar substrates, allowing different hydrogen-bonding pattern in the core domain upon cap closure. This being the case, the geometric modification of AtSgpp3Δ would constrain the substrate ambiguity to a more favorable stereochemistry, accordingly with the kinetic data that reflects the substrate-binding affinity and proper orientation of D-ribose-5-phosphate in the active site of AtSgpp3Δ, over other phosphate ester substrates.

## 5. Conclusions

Summarizing the discussion above, this paper reports the activity profile of the AtSgpp mutant protein AtSgpp3Δ. The deletion of residues Phe-53, Asn-54, and Asn-55 does not affect the pH dependence but the phosphatase activity of AtSgpp3Δ, increasing *K*_m_/*V*_max_ slopes and reducing the value range of *k*_cat_ and the substrate specificity constant *k*_cat_/*K*_m_ of the promiscuous spectrum. Unlike AtSgpp, the lowest affinity was observed for DL-glycerol-3-phosphate, pointing to an essential role of the removed AtGpp counterpart’s residues Phe-53 and Asn-54 in DL-glycerol-3-phosphate chemistry. AtSgpp3Δ displayed highest discrimination *k*_cat_/*K*_m_ ratios than the normal protein, which reflects the substrate-binding affinity and proper orientation in the active site of D-ribose-5-phosphate over the rest of phosphate ester metabolites tested. Not all determinants of AtSgpp’s substrate specificity are known at present—additional geometrical features may also influence the range of sugar phosphates that the enzyme can accommodate. However, our results support the driving role of the cap architecture in the evolutionary biochemistry of the HAD superfamily.

## Figures and Tables

**Table 1:** Kinetic parameter *k*_cat_, *K*_m_, and *k*_cat_/*K*_m_ of the phosphatase activity of purified AtSgpp and deletion mutant protein AtSgpp-ΔF53N54N55 (AtSgpp3Δ) on various organic phosphomonoesters. The reaction mixture contained: 20 mM Tris-HCl (pH 7.0), 5 mM MgCl_2_, 10 mM substrate (different phosphomonoesters) and 25 µg/mL of the purified protein. Reaction temperature: 32 °C. Data represents the mean (95% confidence interval) [standard deviation]. AtSgpp3Δ activity was monitored in parallel to that of the previously reported AtSgpp mutants [15]. The kinetic parameters of the AtSgpp3Δ mutant are therefore compared to the phosphatase activity of the same wild type AtSgpp control.

Substrate	AtSgpp	AtSgpp3Δ
	*k*_cat_ (s^−1^)	
D-ribose-5-phosphate	3.9 (3.6–4.2) × 10 [0.3]	3.1 (2.7–3.5) × 10 [0.4]
2-deoxy-D-glucose-6-phosphate	3.3 (3.2–3.4) × 10 [0.1]	2.3 (2.2–2.4) × 10 [0.1]
D-mannose-6-phosphate	3.2 (2.5–3.9) × 10 [0.6]	1.9 (1.7–2.1) × 10 [0.2]
D-glucose-6-phosphate	2.4 (1.8–3) × 10 [0.5]	1.2 (1.1–1.3) × 10 [0.1]
D-fructose-6-phosphate	1.9 (1.7–2.1) × 10 [0.2]	1.1 (0.8–1.4) × 10 [0.3]
DL-glycerol-3-phosphate	2.3 (2–2.6) × 10 [0.3]	0.7 (0.5–0.9) × 10 [0.2]
	*K*_m_ (M)	
D-ribose-5-phosphate	3.6 (3.5–3.7) × 10^−3^ [0.1]	3.7 (3.5–3.9) × 10^−3^ [0.2]
2-deoxy-D-glucose-6-phosphate	4.6 (4–5.2) × 10^−3^ [0.5]	4.7 (4.5–4.9) × 10^−3^ [0.2]
D-mannose-6-phosphate	4.9 (4.2–5.6) × 10^−3^ [0.6]	6.7 (6.6–6.8) × 10^−3^ [0.1]
D-glucose-6-phosphate	7.1 (6.8–7.4) × 10^−3^ [0.3]	8.3 (8–8.6) × 10^−3^ [0.3]
D-fructose-6-phosphate	7.7 (7.3–8.1) × 10^−3^ [0.4]	10 (9.5–10.5) × 10^−3^ [0.4]
DL-glycerol-3-phosphate	7.3 (7.1–7.5) × 10^−3^ [0.2]	14 (13.7–14.3) × 10^−3^ [0.3]
	*k*_cat_/*K*_m_ (M^−1^s^−1^)	
D-ribose-5-phosphate	1.07 × 10^4^	8.3 × 10^3^
2-deoxy-D-glucose-6-phosphate	7.2 × 10^3^	4.9 × 10^3^
D-mannose-6-phosphate	6.7 × 10^3^	2.8 × 10^3^
D-glucose-6-phosphate	3.4 × 10^3^	1.5 × 10^3^
D-fructose-6-phosphate	2.5 × 10^3^	1.2 × 10^3^
DL-glycerol-3-phosphate	3.1 × 10^3^	0.5 × 10^3^

## Supplementary Materials

The following are available online at https://www.mdpi.com/2079-7737/8/4/77/s1. Supplemental Figure S1. Three-dimensional structure of DL-glycerol-3-phosphatase AtGpp2, phosphosugar phosphatase AtSgpp and the constructed mutant AtSgpp3Δ built using the modelling package MODELLER. Supplemental Figure S2. AtSgpp mutant construction and purification. Supplemental Figure S3. AtSgpp mutant’s phosphatase activity profile. Supplemental Figure S4. AtSgpp mutant’s phosphatase pH dependence.
