# Supplementary material for: Sequence Determinants of Substrate Ambiguity in a HAD Phosphosugar Phosphatase of Arabidopsis Thaliana
Source: Biology (Basel). 2019 Oct 9;8(4):77. doi: 10.3390/biology8040077 (PMC6956230; doi:10.3390/biology8040077)
Supplement: Supplementary file 1 [file biology-08-00077-s001.pdf]

biology

MDPI

Article

Sequence Determinants of Substrate Ambiguity in a HAD Phosphosugar Phosphatase of *Arabidopsis Thaliana*

José A. Caparrós-Martín [1,2,*], Iva McCarthy-Suárez [1] and Francisco A. Culiáñez-Macià [1,†]

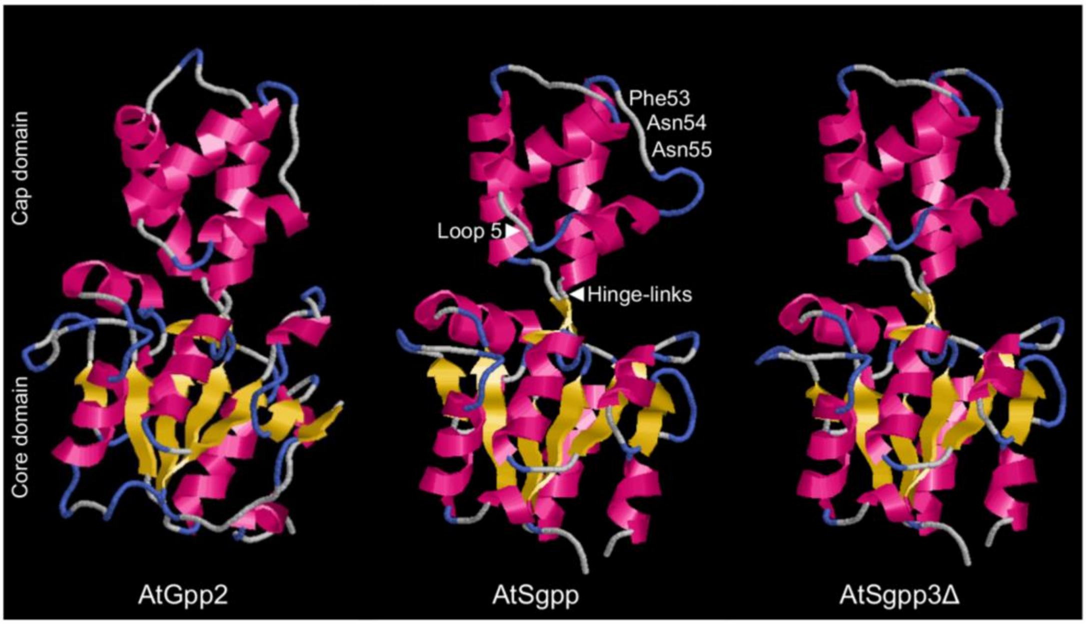

Supplemental Figure S1. Three-dimensional structure of DL-glycerol-3-phosphatase AtGpp2, phosphosugar phosphatase AtSgpp and the constructed mutant AtSgpp3Δ built using the modelling package MODELLER. Deleted residues of the loop in AtSgpp are highlighted. Loop-5 and the hinge that links the cap and core domains are marked with arrowheads.

a

Proteins	Loop	Sequence 5' → 3'
AtSgpp	GFNNGVPID	GGTTTTAACAATGGTGTCCCAATCGAT
AtSgpp3Δ	GGVPID	GGTGGTGTCCCAATCGAT
AtGpp	FNKKFD	TTCAATAAAAAGTTTGAT

b

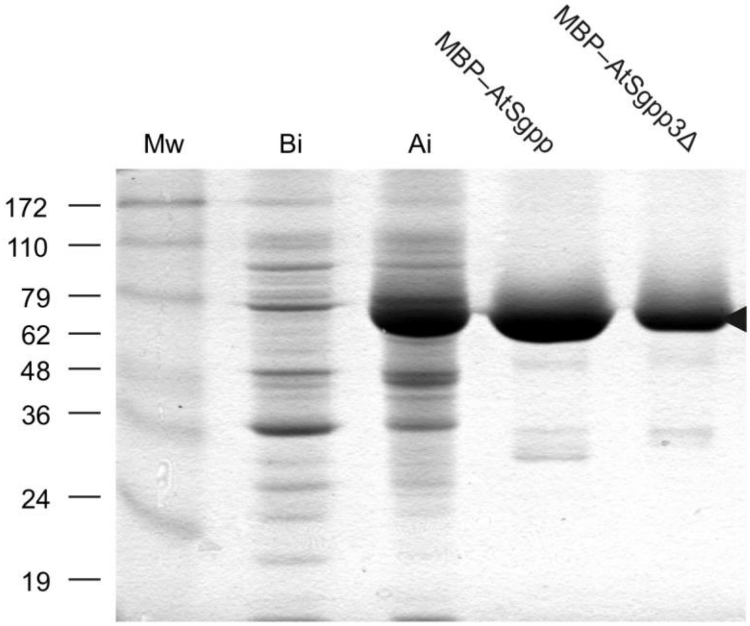

Supplemental Figure S2. AtSgpp mutant construction and purification. (**a,b**) the mutant *AtSgpp* gene was constructed by sequential polymerase chain reaction mutagenesis using appropriate primers and *AtSgpp*-cDNA as template: AtSgpp (phosphosugar phosphatase); AtSgpp3Δ (triple residue deletion mutant ΔF53, ΔN54, ΔN55); AtGpp (DL-glycerol-3-phosphatase) (**a**). (**b**). Denaturing protein electrophoresis of the indicated purified MBP-fused recombinant proteins (arrowhead). Mw, molecular weight ladder (KDa). Lanes labelled as Bi and Ai were loaded with protein extracts from *E.coli* clones to illustrate the corresponding protein profiles before (Bi) and after (Ai) induction of protein expression.

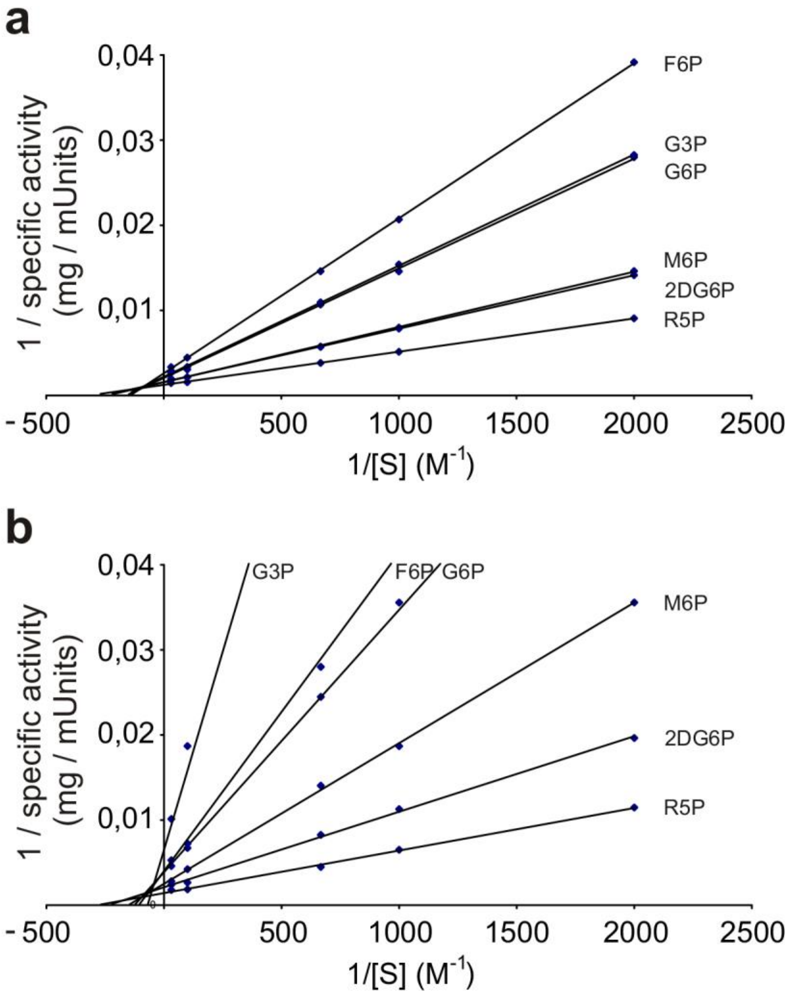

Supplemental Figure S3. AtSgpp mutant's phosphatase activity profile. (**a**,**b**) The apparent *K*m and *V*max for D-ribose-5-phosphate (R5P), 2-deoxy-D-glucose-6-phosphate (2DG6P), D-mannose-6-phosphate (M6P); D-glucose-6-phosphate (G6P), DL-glycerol-3-phosphate (G3P) and D-fructose-6-phosphate (F6P) of the AtSgpp phosphatase (**a**) and AtSgpp3Δ (triple residue deletion mutant ΔF53, ΔN54, ΔN55) (**b**). We evaluated the activity of the AtSgpp3Δ mutant together with the other AtSgpp mutants published earlier [1]. Thus, the reported activity of AtSgpp3Δ is referred to the same *wild type* AtSgpp control values.

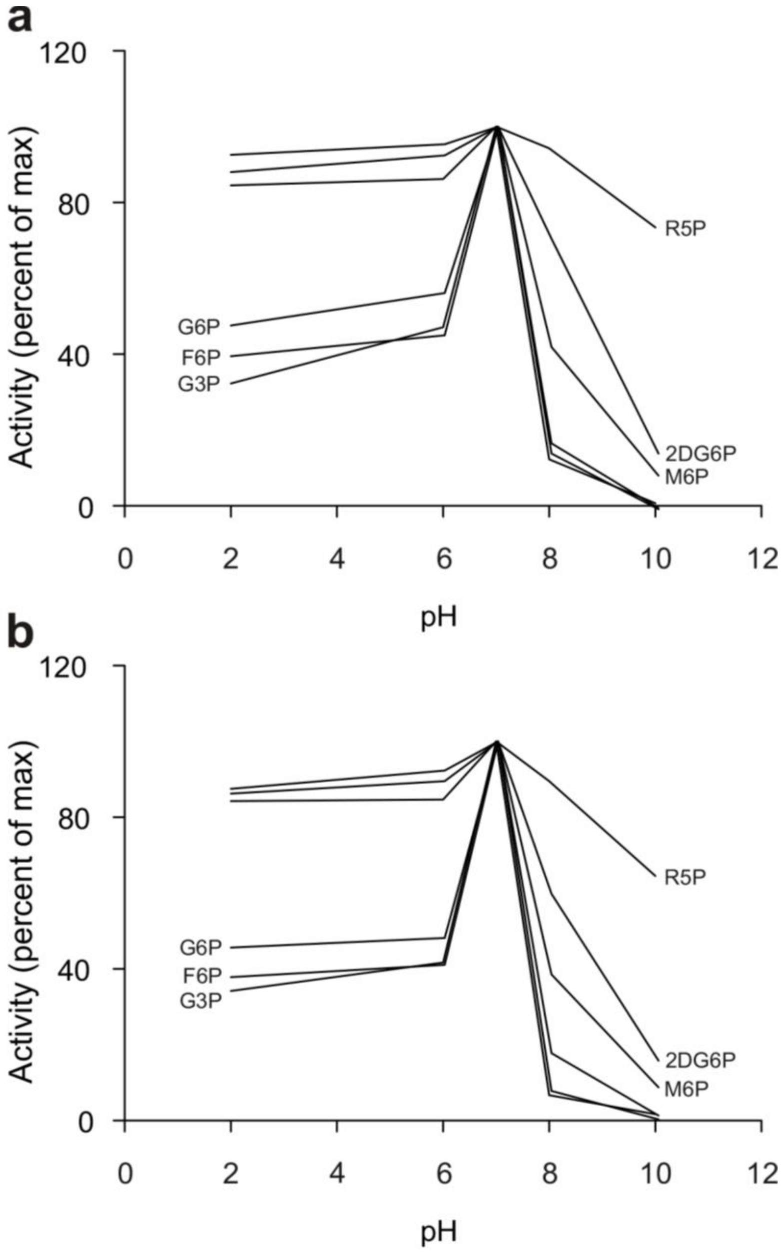

Supplemental Figure S4. AtSgpp mutant's phosphatase pH dependence. (**a**,**b**) Influence of pH on the phosphatase activity for D-ribose-5-phosphate (R5P), 2-deoxy-D-glucose-6-phosphate (2DG6P), D-mannose-6-phosphate (M6P), D-glucose-6-phosphate (G6P), DL-glycerol-3-phosphate (G3P) and D-fructose-6-phosphate (F6P) of the AtSgpp phosphatase (**a**) and AtSgpp3Δ (triple residue deletion mutant ΔF53, ΔN54, ΔN55) (**b**).

Supplemental references

1. Caparros-Martin, J.A.; McCarthy-Suarez, I.; Culianez-Macia, F.A. The kinetic analysis of the substrate specificity of motif 5 in a HAD hydrolase-type phosphosugar phosphatase of Arabidopsis thaliana. *Planta* **2014**, *240*, 479–487, doi:10.1007/s00425-014-2102-6.
